# *TNFA* -308G>A and *IL10* -1082A>G polymorphisms seem to be predictive biomarkers of chronic HCV infection

**DOI:** 10.1186/s12879-021-06835-9

**Published:** 2021-11-03

**Authors:** Angélica Menezes Santiago, Ednelza da Silva Graça Amoras, Maria Alice Freitas Queiroz, Simone Regina Souza da Silva Conde, Izaura Maria Vieira Cayres-Vallinoto, Ricardo Ishak, Antonio Carlos Rosário Vallinoto

**Affiliations:** 1 Laboratory of Virology, Institute of Biological Sciences, Federal University of Pará (Universidade Federal Do Pará - UFPA), Belém, Pará Brazil; 2 João de Barros Barreto Hospital, Federal University of Pará (Universidade Federal do Pará - UFPA), Belém, Pará Brazil; 3 School of Medicine, Institute of Health Sciences, Federal University of Pará (Universidade Federal Do Pará - UFPA), Belém, Pará Brazil; 4 Graduate Program in Virology, Evandro Chagas Institute/SVS/MS, Ananindeua, Pará Brazil

**Keywords:** HCV, TNF-alpha, Interleukin-10, Polymorphism

## Abstract

**Background:**

Genetic changes may induce dysregulated cytokine production and affect the progression of the chronic disease caused by the hepacivirus C (HCV) because the balance of pro- and anti-inflammatory cytokines determines the outcome of infection. This study evaluated the *TNFA* -308G>A and *IL10* -1082A>G polymorphisms in the susceptibility and progress of chronic hepatitis C.

**Method:**

The study included 101 samples from patients with chronic hepatitis C and 300 samples from healthy donors. Polymorphisms were typed by real-time PCR and were analyzed for associations with histopathological parameters (according to METAVIR classification) and HCV viral load.

**Results:**

The polymorphic genotype for the *TNFA* -308G>A variant was not present in the group of patients with chronic hepatitis C and its absence could be associated with protection against HCV infection (p = 0.0477). Patients with the polymorphic genotype of the *IL10* -1082A>G polymorphism had higher HCV viral load than wild-type patients (p = 0.0428). Neither polymorphism was associated with different levels of necroinflammatory activity or fibrosis scores.

**Conclusion:**

Our results suggest the polymorphic genotype at *TNFA* -308G>A as protective against chronic HCV infection, and the polymorphic genotype at the *IL10* -1082A>G variant associated with higher HCV viral load. Further studies must be performed in order to confirm these associations.

## Background

Hepacivirus C (HCV) is the causative agent of hepatitis C and is considered the main cause of liver cancer. It is estimated that over 71 million people are chronically infected by the virus and approximately 399,000 people died from hepatitis C, mostly from cirrhosis and hepatocellular carcinoma [1]. Persistent HCV infection leads to chronic hepatitis, which is mainly due to the inability of the immune system to eliminate the virus [2]. Dysregulated cytokine production is related to the chronicity of hepatitis C; however, no profile of cytokines involved in the development of liver injury has been identified [3].

Tumor necrosis factor alpha (TNF-α) is a pro-inflammatory cytokine that acts both as a mediator of innate immunity and in the cellular immune response. Abnormal TNF-α levels have been associated with chronic HCV infection [4]. Some polymorphisms in the *TNFA* gene are associated with the regulation of cytokine production and coincide with the binding regions of transcription factors [5]. The *TNFA* -308G>A polymorphism has higher transcriptional activity than the wild-type allele [6] and has been associated with different infectious diseases [7–11].

Interleukin (IL)-10 is a potent suppressor of the effector function of T cells, natural killer (NK) cells and, mainly, activated macrophages [8]. Several functional polymorphisms have been described in the promoter region of the *IL10* gene 10 [12], among which the *IL10* -1082A>G polymorphism promotes changes in cytokine levels, with the A allele being related to lower levels and the G allele with higher levels of IL-10 [12]. This polymorphism has been associated with chronic and infectious diseases [13, 14].

The *TNFA* -308G>A and *IL10* -1082A>G polymorphisms have been studied in different groups of patients with HCV infection and no association was reported to the necroinflammatory activity or with fibrosis score [15]. Furthermore, other studies have also found no relationship between these polymorphisms in the *TNF* and *IL10* genes and different stages of the disease [16]. On the other hands, gene variants of cytokines/receptors may influence liver damage in patients chronically infected by HCV genotype [17].

Because the liver is a highly immunotolerant organ, an imbalance of the components related to its suppressor and effector functions may contribute to the persistence of HCV and the progression of chronic cases of hepatitis C. On this background, the present study investigated the influence of the *TNFA* -308G>A and *IL10* -1082A>G polymorphisms on the susceptibility to chronic HCV infection, the progression to different disease stages, and viral persistence. The findings in this study may help to understand the physiology of the biomarkers analyzed and their response to chronic HCV infection as well as the progression to the hepatic diseases.

## Methods

### Study population

The present project was submitted to and approved by the research ethics committees of Santa Casa de Misericórdia do Pará (opinion 772,782/2014) and João de Barros Barreto University Hospital (opinions 962,537/2015 and 2,165,948/2017), in accordance with the principles of the Declaration of Helsinki. All participants were informed about the objectives of the study, and those who agreed to participate signed an informed consent form and answered an epidemiological questionnaire.

The study was carried out by spontaneous demand of patients attended to the liver disease outpatient clinics of the Santa Casa de Misericórdia do Pará Foundation and the João de Barros Barreto University Hospital, including all patients with chronic hepatitis C attended during the study period (May 2014 to April 2016) who met the inclusion criteria and agreed to participate in the study were included. The HCV group consisted of 101 patients, both sexes, treatment naïve, with chronic hepatitis C, characterized by clinical changes, abnormal liver tests and HCV RNA positivity.

The inclusion criteria adopted for the individuals were as follows: age 18 or older, and seropositivity for anti-HCV for more than 6 months and positivity for HCV RNA, as criteria for chronic HCV infection [18], and without antiviral therapy. Individuals coinfected with hepatitis B virus (HBV), hepatitis delta virus, or human immunodeficiency virus (HIV) and patients who used or were using specific antiviral therapy against HCV were excluded from the study.

A control group was formed to compare the genotypic and allelic frequencies of the investigated polymorphisms, which included 300 blood samples from volunteer donors from the Foundation Center for Hemotherapy and Hematology of Pará (Fundação Centro de Hemoterapia e Hematologia do Pará). These volunteers were matched with the patient group for age and sex; were seronegative for HCV, HBV, human immunodeficiency virus 1 (HIV-1), and human T-lymphotropic virus 1/2 (HTLV-1/2).

### DNA extraction

DNA was extracted from peripheral-blood leukocytes using the Puregene kit (Gentra Systems, Minneapolis, Minnesota, USA) according to the manufacturer’s protocol. The procedure included the steps of cell lysis, protein precipitation, DNA precipitation, and DNA hydration.

### Genotyping TNF -308G>A (rs1800629) e IL10 -1082A>G (rs1800896)

Polymorphisms were genotyped by quantitative real-time polymerase chain reaction in the StepOne PLUS Sequence Detector (Applied Biosystems, Foster City, CA, USA). The assay used for each polymorphism contained a pair of primers and a pair of VIC- and FAM-labeled probes for the respective alleles. For both polymorphisms, predesigned and customized TaqMan^®^ SNP Genotyping Assays were used: C_7514879_10 for *TNFA* -308G>A and C_1747360_10 for *IL10* -1082A>G (Thermo Fisher, Carlsbad, California, USA). For each reaction, 2× TaqMan^®^ Universal PCR Master Mix, 1× TaqMan^®^ Assay (diluted from 20×), and 20 ng of DNA was used in a final reaction volume of 10 µL. The following temperature cycle was used for the amplification: 60 °C for 30 s, 95 °C for 10 min, and 50 cycles of 92 °C for 30 s and 60 °C for 1 min and 30 s.

### Complementary exams

All selected patients were clinically evaluated and subjected to a complementary investigation consisting of biochemical (liver enzyme levels: alanine aminotransferase (ALT), aspartate aminotransferase (AST) and gamma-glutamyl transferase (GGT), serological (HBV surface antigen (HBsAg), HBV e antigen (HBeAg), anti-HBeAg, total anti-HBc and anti-HCV), virological (HBV DNA and hepacivirus C RNA), and liver biopsies. These data were transcribed from the medical records into a form developed specifically for this study.

### Histopathological procedures

Liver biopsy specimens were obtained only from patients with medical indications for the investigation of liver parenchyma changes, in compliance with the clinical care protocol. The liver biopsies were performed by a medical professional from one of the study hospitals using a Tru-Cut needle under ultrasound guidance. The sample was sent to the Department of Pathological Anatomy of Federal University of Pará, where they were examined following the department’s routines, which included hematoxylin–eosin (HE), chromotrope aniline blue (CAB), Gomori’s reticulin, and Shikata’s orcein staining.

The histopathological diagnosis followed the METAVIR classification [19], which classifies the activity of the portal and periportal inflammatory infiltrate from 0 to 3 (A0–A3), A0–A1 indicating absent to mild inflammation and A2–A3 indicating moderate to severe inflammation. The structural changes in the liver parenchyma (degree of fibrosis) were classified from 0 to 4 (F0–F4), F0–F1 indicating absent to mild liver fibrosis, F2 indicating moderate liver fibrosis, and F3–F4 indicating liver fibrosis that has progressed to cirrhosis. All data regarding the histopathological profile were obtained from the patients’ medical records.

### Plasma viral load

Plasma viral load was measured by real-time PCR using an Abbott RealTime HCV Amplification Reagent Kit (Abbott Park, Illinois, USA) at the Central Laboratory of the State of Pará (LACEN-PA). HBV viral load quantifications are presented as copies/mL and log10value. The lowest detection level of HBV load was 1.08 log UI/mL, and the highest detection level was 9.00 log UI/mL. HCV viral load quantifications are presented as copies/mL and log10 value. The lowest detection level of HCV load was 1.08 log UI/mL, and the highest detection level was 8.00 log UI/mL.

### Statistical analysis

The information obtained was entered into a database in the Microsoft Office Excel 2013 software. The calculation of the Hardy–Weinberg balance was performed to assess the distribution of genotypic frequencies. The determination of the allelic and genotypic frequencies of the polymorphisms was made by direct counting and the differences between the groups were evaluated using the Chi-square test and the G test. The distribution of HCV viral load levels and biochemical markers between the genotypes of the TNFA -308G>A and IL10 -1082A>G polymorphisms were performed using the Shapiro–Wilk test. From the results of the normality test, non-parametric tests were used (Mann–Whitney test and Kruskal–Wallis test). The Mann–Whitney test was used to assess *TNFA* -308G>A genotypes with HCV viral load and dosage of biochemical markers. The Kruskal–Wallis test was used to analyze *IL10* -1082A>G genotypes in relation to HCV viral load and biochemical markers. Statistical analyses were done with BioEstat software version 5.3, adopting a significance level of p < 0.05.

## Results

Most patients with chronic HCV were male (n = 52; 51%). The median ALT, ASP, and GGT concentrations were 77.9 UI/L, 69.9 UI/L, and 99.6 UI/L, respectively. The median viral load of 5.4 log (Table 1).

**Table 1:** Characterization of patients with chronic HCV

Variable	HCV
Sex (F/M), n (%)	49 (48.5)/52 (51.5)
ALT (IU/L), median/IQR	77.9 ± 58.4
AST (IU/L), median/IQR	69.9 ± 48.6
GGT (IU/L), median/IQR	99.6 ± 95.2
Viral load (log_10_), median/IQR	5.4 ± 0.9
Fibrosis score, n (%)	
0 to 2	67 (66.3%)
3 to 4	34 (33.7%)
Inflammatory activity, n (%)	^a^
0 to 1	54 (60%)
2 to 3	36 (40%)

N: individuals number; HCV: patients with chronic hepatitis C; ALT: alanine aminotransferase (reference value: 16–40 IU/L); AST: aspartate aminotransferase (reference value: 8–54 IU/L); GGT: gamma-glutamyltransferase (reference value: 8–63 IU/L)

^a^Inflammatory activity n = 90

According to the METAVIR classification (Table 1), most patients with chronic HCV had absent to moderate fibrosis, F0–F2 (n = 67; 66.3%). Inflammatory activity was evaluated only in 90 patients because 11 were diagnosed with liver cirrhosis by imaging tests and therefore did not meet the medical indications for biopsy. The majority of the evaluated patients presented with absent or mild inflammatory activity, A0–A1 (n = 54; 60%).

All the genotype frequencies were in Hardy–Weinberg equilibrium. Evaluation of the *TNFA* -308G>A polymorphism (rs1800629) showed that no patient with chronic HCV had the homozygous polymorphic genotype (AA). The comparison of genotypic frequencies showed a significant difference between the HCV group and control group (p = 0.0477). However, no significant differences were found in allelic frequencies (Table 2).

**Table 2:** Genotypic and allelic frequencies of the *TNFA* -308G>A and *IL10* -1082A>G polymorphisms in the study groups

Allelic or genotypic profile	HCV n (%)	CG n (%)	*p*
*TNFA* -308G>A			
GG	82 (81.2)	222 (74)	0.0477*
GA	19 (18.8)	69 (23)	
AA	0 (0)	9 (3)	
*G	0.91	0.86	0.3753**
*A	0.09	0.14	
*IL10* -1082A > G			
AA	62 (61.4)	164 (54.7)	0.4847**
AG	32 (31.7)	109 (36.3)	
GG	7 (6.9)	27 (9)	
*A	0.77	0.73	0.6242**
*G	0.23	0.27	

HCV: patients with chronic hepatitis C; CG: control group

*G-test

**Chi-squared test

At the *IL10* -1082A>G polymorphism (rs1800896), the wild-type genotype and allele (AA and A, respectively) were the most frequent in the HCV and control groups, with no significant differences between the groups (Table 2).

The evaluation of the frequencies of genotypes and alleles of *TNFA* -308G>A and *IL10* -1082A>G in relation to histopathological markers of inflammatory activity and fibrosis score showed no significant differences (Table 3).

**Table 3:** Genotypic and allelic frequencies of the *TNFA* -308G>A and *IL10* -1082A>G polymorphisms in relation to the histopathological aspects of chronic HCV carriers

Genetic profile	Inflammatory activity*			Fibrosis score*		
	0 to 1	2 to 3	*p*	0 to 2	3 to 4	*p*
	n (%)	n (%)		n (%)	n (%)	
*TNFA* -308G>A						
GG	43 (79.6)	30 (83.3)	0.8688	54 (80.6)	28 (82.4)	0.9553
GA	11 (20.4)	6 (16.7)		13 (19.4)	6 (17.6)	
AA	0	0		0	0	
*G	0.90	0.92	0.8048	0.90	0.91	1.000
*A	0.10	0.08		0.10	0.09	
*IL10* -1082A>G						
AA	31 (56.4)	22 (62.9)	0.7530	40 (59.7)	22 (64.7)	0.6839
AG	20 (36.4)	10 (28.6)		23 (34.3)	9 (26.5)	
GG	4 (7.2)	3 (8.5)		4 (6.0)	3 (8.8)	
*A	0.75	0.77	0.8685	0.77	0.78	1.000
*G	0.25	0.23		0.23	0.22	

*G-test

**Fig. 1:**
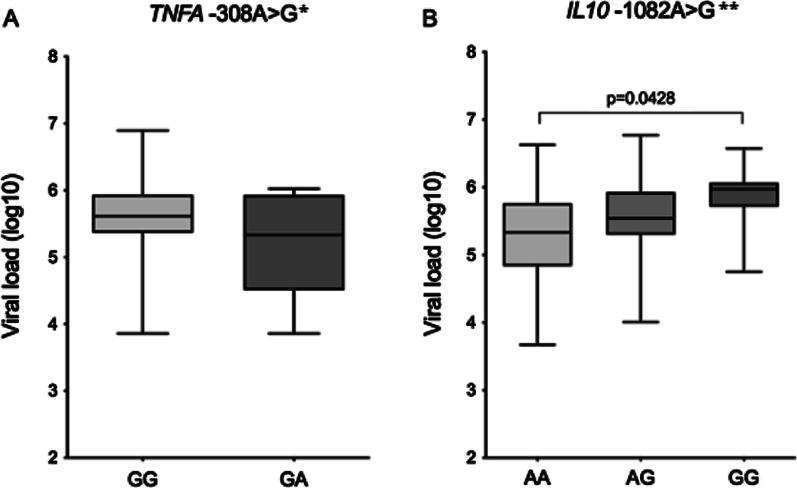
Hepatitis C viral load according the genotype for each polymorphism: **A** *TNFA* -308G>A and **B** *IL10* -1082A>G. * Mann–Whitney test; ** Kruskal–Wallis test

The analysis of viral load showed no significant difference between patients with different *TNFA* -308G>A genotypes. However, patients with the polymorphic genotype of *IL10* -1082A>G had higher HCV viral load than those with the wild-type genotype (p = 0.0428; Fig. 1).

**Fig. 2:**
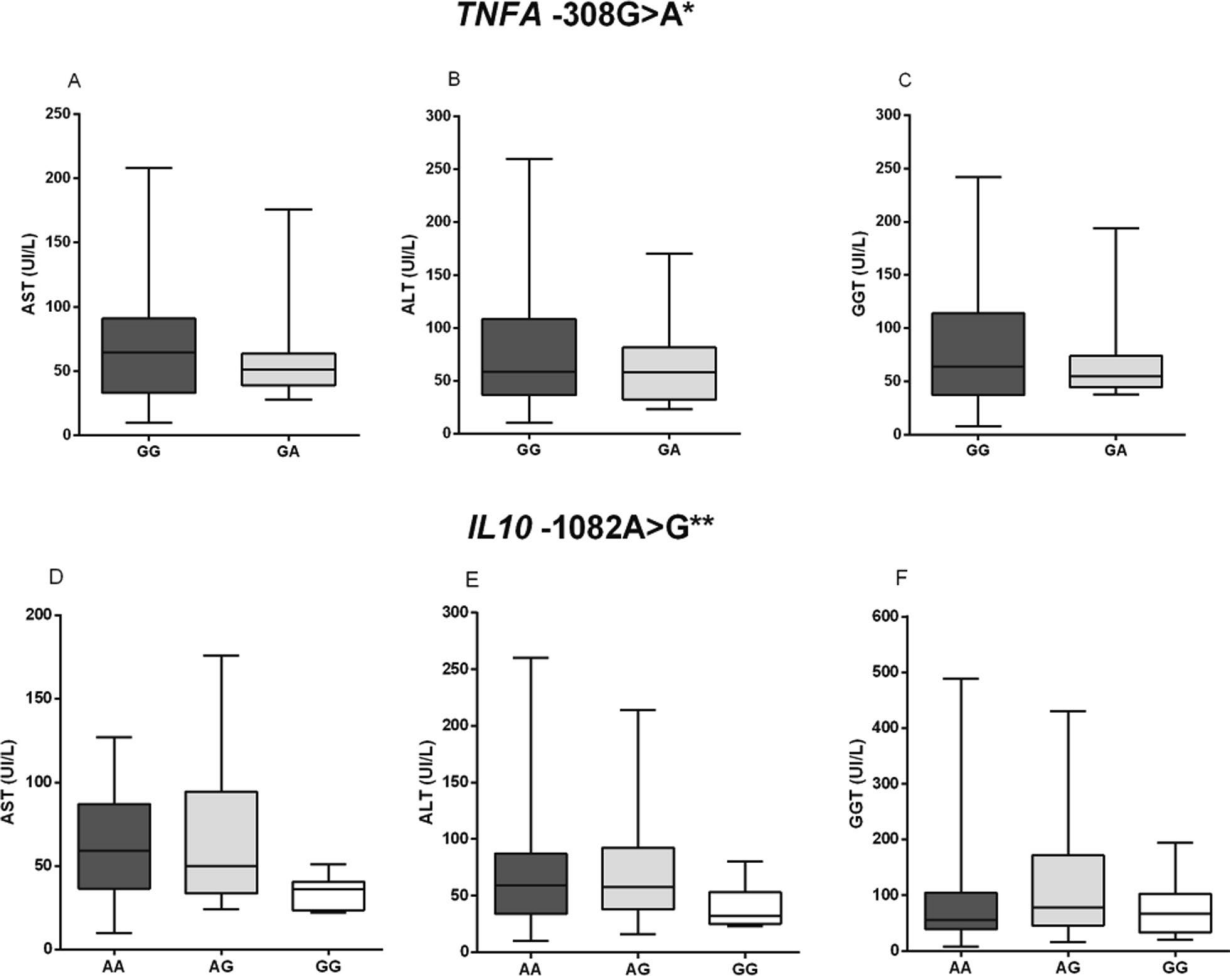
Evaluation of the *TNFA* -308G>A polymorphism according to the levels of **A** AST, **B** ALT and **C** GGT and of the *IL10* -1082A>G polymorphism in relation to the concentrations of **D** AST, **E** ALT and (GGT). All analyzes presented p > 0.05. *Mann–Whitney test; **Kruskal–Wallis test

The evaluation of *TNFA* -308G>A and *IL10* -1082A>G polymorphisms according to the biochemical markers AST, ALT and GGT showed that there was no statistically significant difference (p > 0.05; Fig. 2).

## Discussion

The polymorphic genotype for the *TNFA* -308G>A variant was not present in the group of patients with chronic hepatitis C and its absence could be associated with protection against HCV infection. Patients with the polymorphic genotype of the *IL10* -1082A>G polymorphism had higher HCV viral load than wild-type patients. Neither polymorphism was associated with different levels of necroinflammatory activity or fibrosis scores. There was also no association of polymorphisms with variations in liver enzyme levels.

The course and outcome of HCV infection are determined by its virological characteristics and the immune responses triggered by the virus [20]. HCV is a hepatotropic virus that induces the development of acute and chronic necroinflammatory disease, escaping the immune system in up to 85% of cases [3]. Several cytokines play dual roles in viral infection and are responsible for viral clearance and tissue damage [21].

TNF-α is an important cytokine in the immune response, mediating the inflammatory process through innate immunity pathways and activation of the cellular response, which induces apoptosis or necrosis [22]. Thus, genetic variations in the *TNFA* gene that alter cytokine production levels may contribute to the progression of HCV infection.

In the present study, the polymorphic genotype for the *TNFA* -308G>A variant, correlated with increased expression of the cytokine [6], was not present in the group of patients with chronic HCV. We speculate that the presence of the homozygous variant allele (AA) could contribute to better immune control, preventing the progression of HCV infection. Furthermore, high levels of TNF-α increase the expression of vascular endothelial adhesion molecules and increase the stimulation of endothelial cells and macrophages, which may lead to better infection resolution [21].

The high frequency of the wild-type allele (G) in the group of patients with chronic HCV infection could be a suggestion that in addition to having a higher risk of developing the infection, these patients could have a greater chance of developing the chronic form of the disease, this due to the inadequate production of TNF-α by dendritic cells favors the differentiation of CD4^+^ T cells into IL-10- and non-IFN-γ-producing cells [23]. As the IL-10 cytokine is not effective in resolving the infection it progressed to the chronic form.

Studies on the *TNFA* -308G>A polymorphism performed in other ethnic groups also observed different frequencies of the polymorphic genotype in patients with HCV than without, showing that although the presence of the homozygous polymorphism was not observed in patients from France [24] and in India the prevalence of the polymorphism was higher in the group of patients with chronic HCV [25]. As the population evaluated in this study is trihybrid, formed from the genetic contributions of whites, blacks, and indigenous people [26], the association of the polymorphism with the prevention or risk of HCV infection needs to be better investigated in other ethnic groups. Furthermore, the differences in the results might be due to the methodology used for genotyping the polymorphisms, which were also differents (the present study performed the genotyping by RT-PCR, while the other two used PCR–RFLP and gene sequencing).

IL-10 is an anti-inflammatory cytokine produced by Th2 cells that inhibits the activity of Th1, NK, and macrophage cells, the main cells responsible for pathogen elimination. The cytokine acts by limiting the marked pro-inflammatory response and damage caused by inflammation [8]. In infectious processes, there is a direct correlation between lower IL-10 production and greater disease severity [27].

The *IL10* -1082A>G polymorphism is associated with changes in IL-10 level, the wild-type genotype being associated with lower levels [12]. In the present study, no differences in genotype frequencies were found between the groups with and without HCV infection. The evaluation of this polymorphism in HCV infection by other studies has shown different results. Although the present results were similar to those of another study, which also did not find an association between the frequency of the *IL10* -1082A>G polymorphism and susceptibility to HCV infection [28], Ramos et al. [29] observed that the GG (polymorphic) genotype was associated with increased chances of viral infection resolution. The combined analysis of these results shows that the polymorphism does not influence the protection from or susceptibility to HCV infection but can influence the disease resolution, reducing the chances of progression to the chronic form among those who develop hepatitis C. These differences seem to be associated with differences between ethnic groups, and methodologies used for genotyping the polymorphisms.

The polymorphic genotype (GG) of the *IL10* -1082A>G variant was associated with higher HCV viral load than the wild-type genotype. Most studies that investigated this polymorphism in HCV infection did not assess viral load levels. In the study by Abbas et al. [30], no difference in viral load was observed between genotypes in patients from Pakistan. Viral load has been associated with the frequency of the homozygous genotype (AA) and that of the wild-type (A) allele [16]. The divergence between the results of that study and the present study may be related to the type of analysis performed, Gao et al. [16] evaluated the frequencies of genotypes in relation to the presence or absence of HCV RNA, while the present study evaluated the absolute plasma levels, which were converted to their base-10 logarithm. In addition, the differences may also be related to the ethnicity of the populations assessed between the different studies. The population evaluated in this work is originally from the Brazilian Amazon and has a genetic contribution from Europeans, native Indians and Africans [26], which could contribute to the result found. Some studies have shown that the genetic influence of ethnicity is associated with variations in genes related to the individual’s response to diseases [31, 32].

The polymorphic genotype (GG) for *IL10* -1082A>G is associated with higher IL-10 expression. This cytokine inhibits the activation of Th1, NK, and macrophage cells, which are the main cells responsible for the elimination of HCV; higher levels of IL-10 reduce inflammatory activity at the infection site, favoring the persistence of the virus in the tissue, the main characteristic of chronic infection [2]. Our findings raise the hypothesis that the polymorphic genotype could favor an increase in viral load in the liver tissue; but follow-up studies, as well as mechanistic studies, are needed to confirm this hypothesis.

The *TNFA* -308G>A and *IL10* -1082A>G polymorphisms were not associated with different levels of necroinflammatory activity or with fibrosis score. Several studies have also found no relationship between these polymorphisms in the *TNF* and *IL10* genes and different stages of the disease [15, 16, 24, 28, 33]; probably because the disease progression is due to other factors and not only to the host genetic profile.

Although the TNFA -308G>A and IL10 -1082A>G polymorphisms are associated with variations in the levels of the TNF-α and IL-10 cytokines, it was not possible to directly assess this relationship in the present study. Due to financial limitations, plasma cytokine dosage was not performed, which was the limitation of the study.

In conclusion, the polymorphic genotype at *TNFA* -308G>A was not present in the group of patients with chronic hepatitis C, and even though it could represent a protective action of this SNP against HCV infection, we understand that further studies must be performed in order to confirm it. In the same way, considering that this was a cross-sectional study, the polymorphic genotype for variant *IL10* -1082A>G need to be better analyzed in a follow-up study in order to confirm its association with viral persistence.

## Data Availability

Data are available upon request from the corresponding author.

## Abbreviations

HCV: Hepacivirus C
TNF-α: Tumor necrosis factor alpha
IL-10: Interleukin (IL)-10
NK: Natural killer
HBsAg: Hepatitis B surface antigen
HBeAg: Hepatitis B e antigen
Anti-HBeAg: Hepatitis B e antigen antibody
Anti-HBc: Hepatitis B core antibody
Anti-HCV: Hepacivirus C antibody
RNA: Ribonucleic acid
HBV: Hepatitis B virus
HIV-1: Human immunodeficiency virus 1
HTLV-1/2: Human T-lymphotropic virus 1/2
ALT: Alanine aminotransferase
AST: Aspartate aminotransferase
GGT: Gamma-glutamyl transferase

## References

[1] World Health Organization. Hepatitis C. https://www.who.int/news-room/fact-sheets/detail/hepatitis-c.

[2] Chigbu DI, Loonawat R, Sehgal M, Patel D, Jain P (2019). Hepatitis C virus infection: host–virus interaction and mechanisms of viral persistence. Cells.

[3] Fallahi P, Ferri C, Ferrari SM, Corrado A, Sansonno D, Antonelli A (2012). Cytokines and HCV-related disorders. Clin Dev Immunol.

[4] Kusumoto K, Uto H, Hayashi K, Takahama Y, Nakao H, Suruki R (2006). Interleukin-10 or tumor necrosis factor-alpha polymorphisms and the natural course of hepatitis C virus infection in a hyperendemic area of Japan. Cytokine.

[5] Abraham LJ, Kroeger KM (1999). Impact of the -308 TNF promoter polymorphism on the transcriptional regulation of the TNF gene: relevance to disease. J Leukoc Biol.

[6] Kroeger KM, Carville KS, Abraham LJ (1997). The -308 tumor necrosis factor-alpha promoter polymorphism effects transcription. Mol Immunol.

[7] Cabrera M, Shaw MA, Sharples C, Williams H, Castes M, Convit J, Blackwell JM (1995). Polymorphism in tumor necrosis factor genes associated with mucocutaneous leishmaniasis. J Exp Med.

[8] Couper KN, Blount DG, Riley EM (2008). IL-10: the master regulator of immunity to infection. J Immunol.

[9] Eskandari-Nasab E, Moghadampour M (2018). The relationship between IFN-γ and TNF-α gene polymorphisms and brucellosis: a meta-analysis. Adv Clin Exp Med..

[10] Eskdale J, Gallagher G, Verweij CL, Keijsers V, Westendorp RG, Huizinga TW (1998). Interleukin 10 secretion in relation to human IL-10 locus haplotypes. Proc Natl Acad Sci USA.

[11] Pabalan N, Chaisri S, Tabunhan S, Tarasuk M, Jarjanazi H, Steiner T (2017). Associations of tumor necrosis factor-α-308 polymorphism with dengue infection: a systematic review and meta-analysis. Acta Trop.

[12] Turner DM, Williams DM, Sankaran D, Lazarus M, Sinnott PJ, Hutchinson IV (1997). An investigation of polymorphism in the interleukin-10 gene promoter. Eur J Immunogenet.

[13] Lesiak A, Zakrzewski M, Przybyłowska K, Rogowski-Tylman M, Wozniacka A, Narbutt J (2014). Atopic dermatitis patients carrying G allele in -1082 G/A IL-10 polymorphism are predisposed to higher serum concentration of IL-10. Arch Med Sci.

[14] Meenakshi P, Ramya S, Shruthi T, Lavanya J, Mohammed HH, Mohammed SA (2013). Association of IL-1β +3954 C/T and IL-10-1082 G/A cytokine gene polymorphisms with susceptibility to tuberculosis. Scand J Immunol.

[15] Talaat RM, Esmail AA, Elwakil R, Gurgis AA, Nasr MI (2012). Tumor necrosis factor-alpha -308G/A polymorphism and risk of hepatocellular carcinoma in hepatitis C virus-infected patients. Chin J Cancer.

[16] Gao QJ, Liu DW, Zhang SY, Jia M, Wang LM, Wu LH, Wang SY, Tong LX (2009). Polymorphisms of some cytokines and chronic hepatitis B and C virus infection. World J Gastroenterol.

[17] Moreira ST, Silva GF, de Moraes CF, Grotto RM, de Moura Campos Pardini MI (2016). Influence of cytokine and cytokine receptor gene polymorphisms on the degree of liver damage in patients with chronic hepatitis C. Meta Gene.

[18] Brasil. Ministério da Saúde. Secretaria de Vigilância em Saúde. Departamento de DST, Aids e Hepatites Virais. Protocolo clínico e diretrizes terapêuticas para hepatite viral C e coinfecções, 2011. 144 p. Técnicos). https://bvsms.saude.gov.br/bvs/publicacoes/protocolos_diretrizes_hepatite_viral_c_coinfeccoes.pdf.

[19] Bedossa P, Poynard T (1996). An algorithm for the grading of activity in chronic hepatitis C. The METAVIR Cooperative Study Group. Hepatology.

[20] Shin EC, Sung PS, Park SH (2016). Immune responses and immunopathology in acute and chronic viral hepatitis. Nat Rev Immunol.

[21] Guidotti LG, Chisari FV (2001). Noncytolytic control of viral infections by the innate and adaptive immune response. Annu Rev Immunol.

[22] Holbrook J, Lara-Reyna S, Jarosz-Griffiths H, McDermott M (2019). Tumour necrosis factor signalling in health and disease. F1000Res.

[23] Boks MA, Kager-Groenland JR, Mousset CM, van Ham SM, ten Brinke A (2014). Inhibition of TNF receptor signaling by anti-TNFα biologicals primes naïve CD4(+) T cells towards IL-10(+) T cells with a regulatory phenotype and function. Clin Immunol.

[24] Bouzgarrou N, Hassen E, Gabbouj S, Schvoerer E, Ben Mami N, Triki H (2010). Lack of effect of tumor necrosis factor-alpha -308 G/A polymorphism on severity of liver fibrosis in Tunisian hepatitis C virus (HCV)-infected patients. Gastroenterol Clin Biol.

[25] Dogra G, Chakravarti A, Kar P, Chawla YK (2011). Polymorphism of tumor necrosis factor-α and interleukin-10 gene promoter region in chronic hepatitis C virus patients and their effect on pegylated interferon-α therapy response. Hum Immunol.

[26] Santos NP, Ribeiro-Rodrigues EM, Ribeiro-Dos-Santos AK, Pereira R, Gusmão L, Amorim A, Guerreiro JF, Zago MA, Matte C, Hutz MH, Santos SE (2010). Assessing individual interethnic admixture and population substructure using a 48-insertion-deletion (INSEL) ancestry-informative marker (AIM) panel. Hum Mutat.

[27] Ouyang W, Rutz S, Crellin NK, Valdez PA, Hymowitz SG (2011). Regulation and functions of the IL-10 family of cytokines in inflammation and disease. Annu Rev Immunol.

[28] Bouzgarrou N, Hassen E, Farhat K, Bahri O, Gabbouj S, Maamouri N (2009). Combined analysis of interferon-gamma and interleukin-10 gene polymorphisms and chronic hepatitis C severity. Hum Immunol.

[29] Ramos JA, Silva R, Hoffmann L, Ramos AL, Cabello PH, Urményi TP (2012). Association of IL-10, IL-4, and IL-28B gene polymorphisms with spontaneous clearance of hepatitis C virus in a population from Rio de Janeiro. BMC Res Notes.

[30] Abbas Z, Moatter T, Hussainy A, Jafri W (2005). Effect of cytokine gene polymorphism on histological activity index, viral load and response to treatment in patients with chronic hepatitis C genotype 3. World J Gastroenterol.

[31] Pinto P, Salgado C, Santos NP, Santos S, Ribeiro-dos-Santos A (2015). Influence of genetic ancestry on INDEL markers of NFKb1, CASP8, PAR1, IL4 and CYP19A1 genes in leprosy patients. PLoS Negl Trop Dis.

[32] Pontoriero AC, Trinks J, Hulaniuk ML, Caputo M, Fortuny L, Pratx LB (2015). Influence of ethnicity on the distribution of genetic polymorphisms associated with risk of chronic liver disease in South American populations. BMC Genet.

[33] Sheneef A, Esmat MM, Mohammad AN, Mahmoud AA, Moghazy HM, Noureldin AK (2017). Interleukin-10 and interferon gamma gene polymorphisms and hepatitis C virus-related liver cirrhosis risk. J Interferon Cytokine Res.

